# Analysis of Risk Factors for Early Progression of Prostate Cancer After Initial Endocrine Therapy

**DOI:** 10.7150/jca.81513

**Published:** 2023-02-13

**Authors:** Bowen Hu, Feng Shu, Yan Liu, Jiaying Zhu, Haojie Wang, Nengqing Xie, Xiaoling Liu, Guanmin Jiang, Minbo Yan, Yingbo Dai

**Affiliations:** 1 Department of Urology, the Fifth Affiliated Hospital of Sun Yat-sen University, Zhuhai, China.; 2 Department of Clinical laboratory, the Fifth Affiliated Hospital of Sun Yat-sen University, Zhuhai, China.; 3 Department of Pediatrics, the Inner Mongolia Maternal and Child Health Hospital, Hohhot, China.

**Keywords:** prostate cancer, hormone therapy, tumour markers, prognostic factors.

## Abstract

**Background:** Prolonged androgen deprivation therapy (ADT) in patients with prostate cancer can eventually lead to the development of castration-resistant prostate cancer (CRPC). Once CRPC occurs, the patient's prognosis will be inferior. However, the risk factors for progression to CRPC in a short period of time are unclear.

**Methods:** We retrospectively analyzed prostate cancer patients who received their first ADT between January 1, 2015 and January 1, 2021. The main statistical methods used were a logistic regression model and Kaplan-Meier survival analysis.

**Results:** Among 159 prostate cancer patients initially treated with ADT, 90 were screened for inclusion. Patients who progressed to CRPC after ADT were included in group B and others were included in group A. Group B was divided into group B1 and B2 according to whether CRPC progressed within 18 months. Multi-factor logistic regression analysis showed that the time to PSA nadir (TTN) (p = 0.031) and serum lactate dehydrogenase (LDH) (p = 0.013) were significantly different between Group A and B. TTN (p < 0.001), LDH (p = 0.001) and platelet to lymphocyte ratio (PLR) (p = 0.005) were significantly different between Group B1 and B2. Kaplan-Meier survival analysis and log-rank tests showed that TTN, LDH, and PLR statistically differed in CRPC patients' progression-free survival. The ROC curve showed the AUC value of TTN combined with PLR and LDH increased to 0.958 (95% CI 0.911-0.997, p < 0.001). The Chi-square test showed that the expression of p63 in group A was higher than that in groups B1 (p = 0.002) and B2 (p = 0.001).

**Conclusion:** Lower TTN, higher LDH and PLR were associated with early CRPC occurrence after ADT in hormone-sensitive prostate cancer patients. p63 expression was associated with favorable prognosis in prostate cancer patients.

## Introduction

Prostate cancer is one of the most common cancers in humans and the fifth leading cause of cancer deaths in the United States 1. In 2022, there will be 268,490 new cases of prostate cancer, ranking first in the total number of cancers in men in the United States, and 34,500 prostate cancer deaths, ranking second in the total number of cancer deaths in men in the United States, according to the latest study 2. Although surgical resection is commonly used to improve patient prognosis, it is limited to patients with early-stage prostate cancer. However, prostate cancer is markedly heterogeneous, with some patients (10.7%) already having metastases at the time of diagnosis of prostate cancer 3. Radical surgical treatment is often minimally effective and extremely risky in these patients.

For advanced prostate cancer, the current treatment options are endocrine therapy, chemotherapy, radiotherapy, targeted therapy, and immunotherapy. In recent years, the rapid development of targeted therapy and immunotherapy has significantly improved the prognosis of patients with advanced prostate cancer. Still, there is no doubt that endocrine therapy remains one of the primary treatment options for advanced prostate cancer. In particular, the remarkable results of large-scale clinical trials related to novel endocrine therapeutic agents such as abiraterone and enzalutamide in the last decade have widely promoted the clinical research and application of endocrine therapy for prostate cancer 4,5.

It has long been known that prostate cancer is androgen-dependent for growth and development, and androgen deprivation is an effective treatment strategy widely used in clinical care 6. Prostate cancer endocrine therapy, also known as androgen deprivation therapy (ADT), is a milestone in the history of prostate cancer treatment. Since the early 1940s, when Huggins et al. 7 were the first to demonstrate that ADT could effectively slow the progression of prostate cancer, ADT has evolved from an adjuvant treatment strategy to alleviate metastatic symptoms to a currently accepted first-line treatment option for advanced prostate cancer.

Patients with prostate cancer generally respond well to endocrine therapy. Still, after one to two years of treatment, disease recurrence mainly occurs, at which point the disease enters the stage of castration resistance and becomes castration-resistant prostate cancer (CRPC) 8. Once CRPC occurs, patients will face problems such as disease progression, decreased quality of life, and shortened survival 9. Therefore, there is an urgent need to find risk or protective factors for relevant clinical indicators to implement early prevention measures, extend the time from initial ADT to diagnosis of CRPC, or predict the time of CRPC occurrence in advance to guide clinical treatment.

Previous similar studies have mostly focused on clinical data and neglected to combine laboratory and pathological data. The rich and accessible aboratory data collection can lead to early diagnosis and clinical treatment of diseases. Pathology is the recognized gold standard for disease diagnosis. Pathological data can make a clear diagnosis and provide accurate information for the severity and prognosis of the disease. Based on the collection of clinical data, this study focused on analyzing the hematological and immunohistochemical indexes of prostate cancer patients before ADT. In order to explore the risk factors of hormone-sensitive prostate cancer (HSPC) progressing to CRPC in a short time, this study was conducted in two steps. The first part explores the risk factors of HSPC progressing to CRPC and the second part analyses why HSPC went to CRPC in the early stage. Therefore, based on predicting the risk factors for HSPC progression, a simple exploration of the time of progression to CRPC makes this study richer and more reasonable.

## Materials and Methods

### Patients and our study

**Figure 1:**
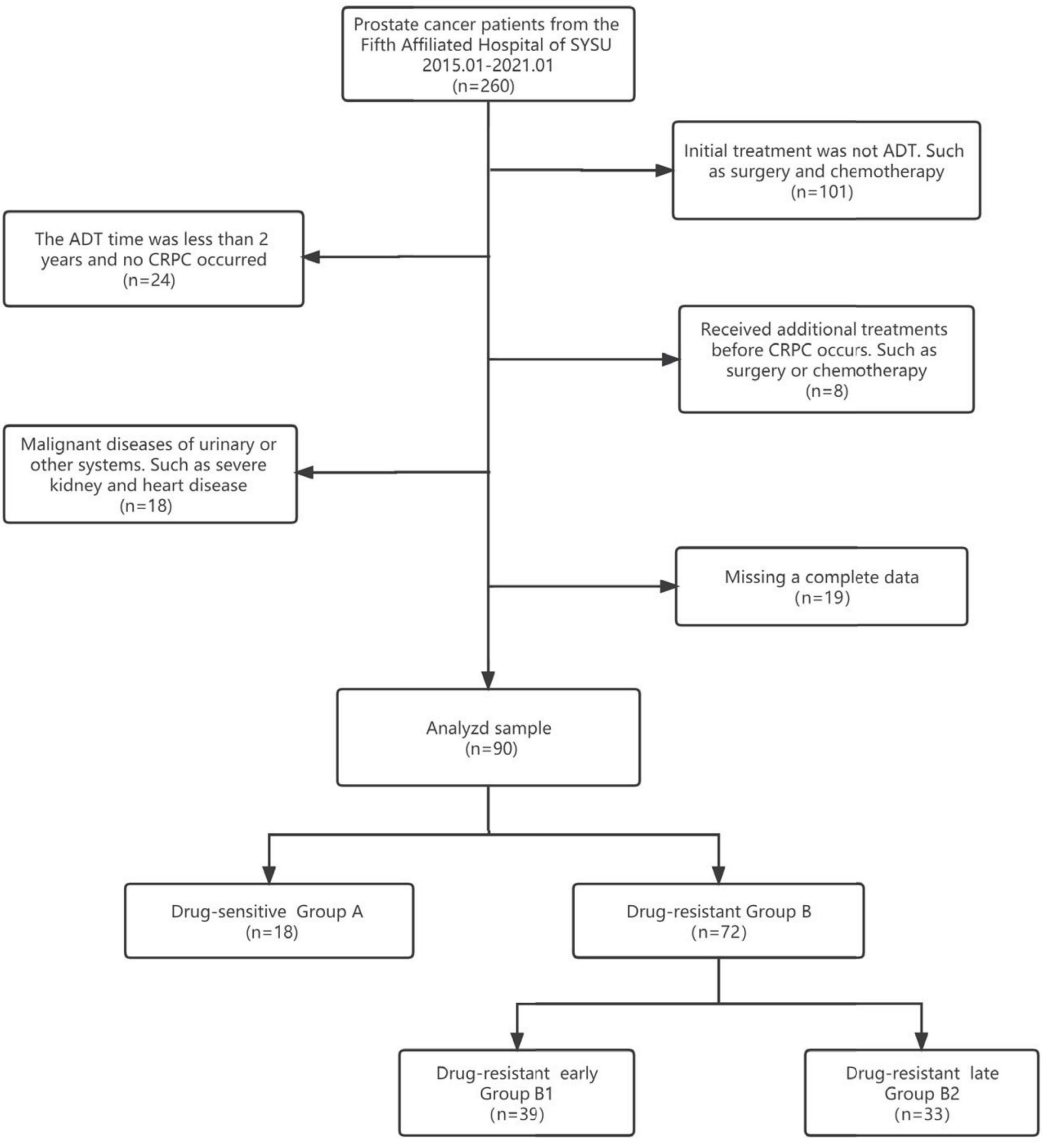
Data selection and grouping process flow chart.

Clinical information from patients with pathologically diagnosed prostate adenocarcinoma who were initially treated with ADT from January 1, 2015, to January 1, 2021, was extracted from the clinical health information system (HIS) of the Fifth Affiliated Hospital of Sun Yat-sen University. All procedures performed in this study followed the Declaration of Helsinki (as revised in 2013) and were approved by the Ethics Committee of the fifth affiliated hospital of Sun Yat-sen University (No.: 2022#K01-1). Because of the retrospective nature of the research, we waived the requirement for informed consent. Patients received regular follow-ups every 3 to 6 months after the start of ADT, underwent drug castration (triptorelin or goserelin), and took anti-androgen drugs (bicalutamide or flutamide). All patients were randomised to receive one of triptorelin or goserelin and one of bicalutamide or flutamide. The expected patient survival time was >24 months. The flow chart of data selection and the grouping process is shown in Figure 1. Compared with previous studies, the data of our center was unique in that "a large proportion of HSPC progressed to CRPC in a short time". According to this feature, we grouped CRPC by time to find the risk factors for developing CRPC quickly. We defined group A as "length of ADT treatment ≥ 24 months and no CRPC until the end of follow-up". We combined the data we collected with previous studies 9,10, Group B1 was defined as progression to CRPC in a short period (≤18 months), and group B2 was defined as progression to CRPC in a relatively long period (> 18 months).

According to the 2020 revised guidelines of the European Association of Urology (EAU) 11, the diagnostic criteria for determining CRPC are as follows: serum testosterone reaches castration level (serum testosterone <50 ng/dL or 1.7 nmol/L); and biochemical recurrence (3 consecutive times, at least 1 week apart, and PSA elevation at least 50% above minimum value), or imaging progress (2 or more new bone metastases found on bone scans, or compared with before endocrine therapy, bone metastasis is from scratch, or magnetic resonance imaging suggests the expansion of soft tissue invasion in prostate cancer). The time of the first rise of PSA after ADT was taken as the occurrence time point of CRPC 12.

### Data collection

We collected clinical data, haematological test indicators and immunohistochemical markers before endocrine treatment of patients with prostate cancer, including patient age, prostate-specific antigen (PSA) level at the commencement of ADT, the time to PSA nadir (TTN) value after ADT, the rate of PSA decline, the tumour, node, metastasis (TNM) stage of prostate cancer, presence of bone metastasis, and the pathological Gleason score (GS) of prostate cancer. We collected common detection indicators, including white blood cell count (WBC), red blood cell count (RBC), haemoglobin concentration (Hb), platelets count (PLT), hematocrit (HCT), mean corpuscular volume (MCV), mean corpuscular haemoglobin (MCH), mean corpuscular haemoglobin concentration (MCHC), absolute neutrophil count (ANC), lymphocyte count (LY), platelet to lymphocyte ratio (PLR), neutrophil to lymphocyte ratio (NLR), monocyte count (MONO), procalcitonin (PCT), mean platelet volume (MPV). We also collected the haematological liver function detection indicators, including alanine transaminase (ALT), aspartate aminotransferase (AST), aspartate aminotransferase/alanine transaminase (AST/ALT) and lactate dehydrogenase (LDH). Immunohistochemical markers of pathological tissue included AR, PSA, PSAP, CK, p504s and p63.

### Follow-up

All patients were followed up every 3 to 6 months for 3 to 60 months, with an average period of 24.75 months. The minimum follow-up time for Group A was 24 months, the maximum follow-up time was 60 months, the mean follow-up time was 33.78 months, and the median follow-up time was 33 months. No patients in group A developed CRPC during follow-up. In Group B, the minimum follow-up time was 3 months, the maximum follow-up time was 59 months, the mean follow-up time was 19.21 months, and the median follow-up time was 17 months. During follow-up, all patients in group B progressed to CRPC. Physical examination, PSA testing, testosterone testing, routine blood examination and chemical analysis, chest radiographs, pelvic computer tomography, or MRI were performed at each follow-up. The study endpoint for each patient was the occurrence of CRPC or the date of the last follow-up. Progression time i.e., CRPC-free survival, was the interval from the start of endocrine therapy to the event of CRPC or the date of the last follow-up.

### Statistical analysis

Statistical analysis was performed using the SPSS v. 25.0 (Chicago, IL, USA) statistical software package and MedCalc v.17.1. T-test was used for measurement data conforming to normal distribution and homogeneity of variance, and rank sum test was used for others. The chi-square tested categorical variables, and the rank sum was tested for rank data. A logistic regression model was applied to analyze and assess the effects of these factors on progression and early progression to CRPC. After converting statistically significant count data for multivariate analysis into categorical variables, the relationship between these risk factors and time to advance to CRPC was further verified using Kaplan-Meier survival analysis and the log-rank test. We examined predicted performances with Receiver Operator Characteristic (ROC) Curves. Correlations were tested using Pearson's correlation analysis. P values involving multiple comparisons in the text had been corrected using the Benjamini and Hochberg methods.

## Results

### Analysis of risk factors for progression to CRPC after endocrine therapy

A total of 90 patients were enrolled in the study, 18 in Group A and 72 in Group B. We defined Group A as "length of ADT treatment ≥24 months and no CRPC until the end of follow-up". The mean age of the drug-sensitive group was 70.72 ± 8.94 years and of the drug-resistant group was 70.67 ± 9.64 years (Table 1). In univariate analysis, PSA before ADT (p = 0.003), PSA nadir (p = 0.023), TTN (p < 0.001), rate of PSA decline (p < 0.001), N stage (p = 0.003), M stage (p < 0.001), bone metastases (p = 0.011), GS (p = 0.032), Hb (p = 0.030), and LDH (p < 0.001) all showed statistical significance (p < 0.05) (Table 2).

Multi-factor logistic regression analysis incorporated variables that are statistically different from univariate analysis and those that have been previously reported as significant in the literature (PLR, NLR, AST). The results of the research showed that TTN (Odds Ratio [OR] = 0.339 95%, CI (0.127-0.905), p = 0.031) was a protective factor for progression to CRPC.LDH (OR = 1.065, 95% CI (1.014-1.119), p = 0.013) was a risk factor for progression to CRPC in patients with HSPC (Table 2).

### Analysis of risk factors for early progression to CRPC after endocrine therapy

There were 72 people in the drug-resistant group, including 39 in Group B1 and 33 in Group B2. The difference between Group B1 and B2 was the time to progression to CRPC. Group B1 was defined as progression to CRPC in a short period (≤ 18 months), and Group B2 was defined as progression to CRPC in a relatively long period (> 18 months). The mean age in the early-onset drug-resistant group was 69.92 ± 10.06 years, and the mean age in the late-onset drug-resistant group was 71.55 ± 9.18 years (Table 3). In the univariate analysis of Group B1 and B2, PSA nadir (p = 0.023), TTN (p < 0.001), WBC (p = 0.007), PLT (p = 0.026), MCV (p = 0.016), MCHC (p = 0.030), PLR (p = 0.044), PCT (p= 0.015) and LDH (p= 0.019) were significantly different (p < 0.05) (Table 4).

Multi-factor logistic regression analysis aslo incorporated variables that are statistically different from univariate analysis and those that have been previously reported as significant in the literature (PLR, NLR, AST). The results of the study showed that TTN (OR = 0.548, 95% CI (0.441-0.732), p < 0.001) was a protective factor for the time of progression of CRPC. PLR (OR = 1.016, 95% CI (1.015-1.028), p= 0.005) and LDH (OR = 1.018, 95% CI (1.007-1.029), p= 0.001) were risk factors for early progression to CRPC (Table 4). We also compared the variability of TTN and LDH in Group A with Group B1 and B2 using the Kruskal-Wallis H test, and the final results showed: In Group A vs Group B1, TTN (H = 52.035, p < 0.001), LDH (H = -41.037, p < 0.001) were statistically different. In Group A vs Group B2, TTN (H = 22.358, p < 0.001) and LDH (H = -28.768, p < 0.001) were statistically different as well (Table 5).

### The expression of each receptor in different groups

We analyzed the immunohistochemical results of 90 patients before ADT. Due to the deletion of missing data, the following indicators were selected for analysis (AR, PSA, PSAP, CK, p504s, p63). Our study showed that AR, PSA, PSAP, CK and p504s receptors were highly expressed among Group A, B1, and B2, especially AR and PSAP receptors (Table 6). The expression of p63 was half in Group A, but significantly lower in Group B1 and B2. Chi-square test showed that the expression of p63 in Group A was significantly different from that in Group B (χ^2^ = 14.214, p = 0.001), B1(χ^2^ = 9.188, p = 0.002) and B2(χ^2^ = 10.833, p = 0.001) (Table 7). There was no significant difference in the expression of AR, PSA, PSAP, CK, and p504s among the groups.

### Relationship between TTN, LDH, and PLR, and time of progression of CRPC

**Figure 2:**
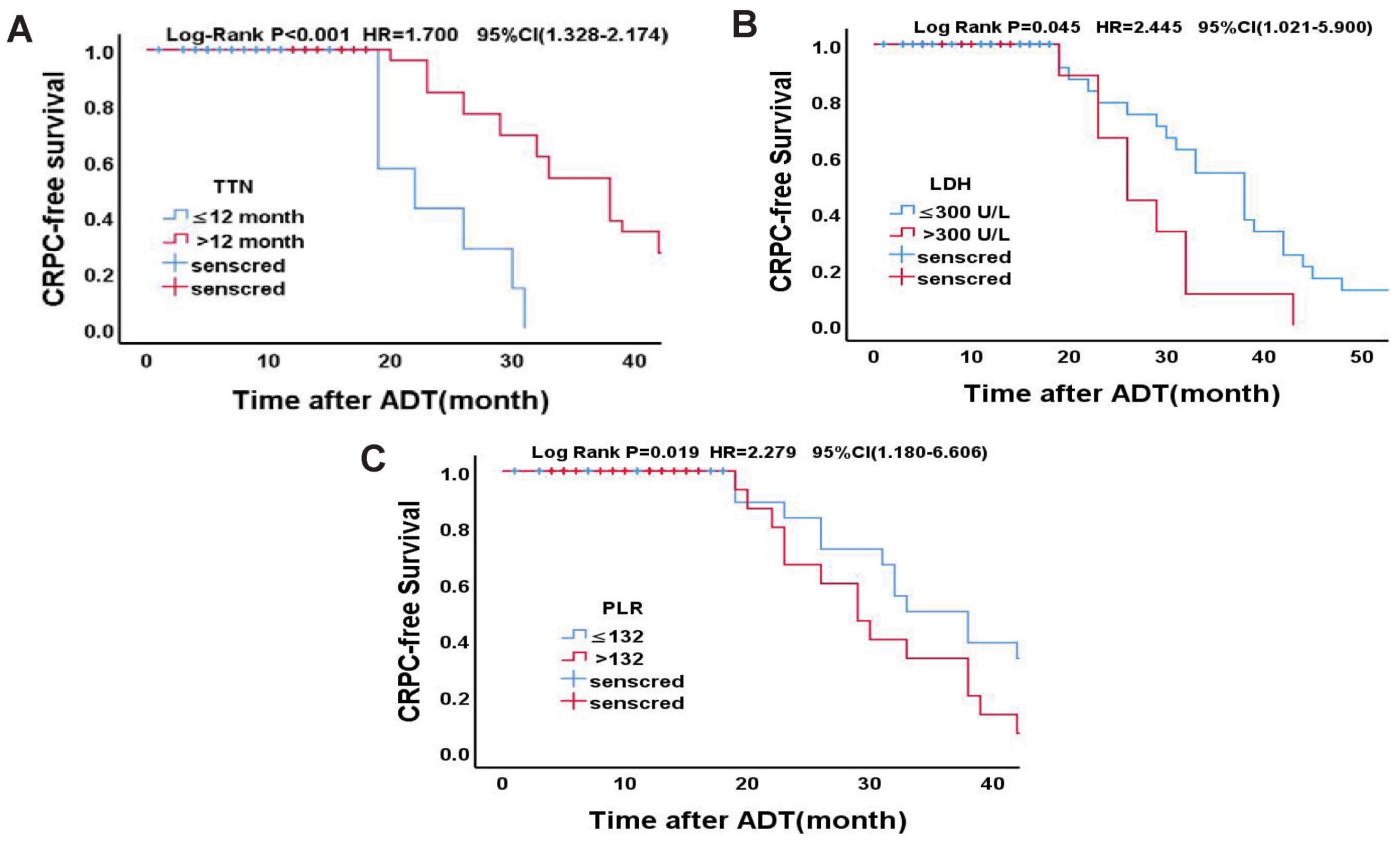
** Kaplan-Meier analysis for CRPC-free survival in Drug-resistant Group. (A)**TTN (≤12 months vs. >12 months); **(B)**LDH (≤300U/L vs. >300U/L); **(C)**PLR (≤132 vs. >132)

Statistically significant count data for the multivariate analysis were transformed into categorical variables and were further validated using the Kaplan-Meier survival analysis and the log-rank test. According to the cut-off value of TTN in a previous study 13, the median time to endpoint was 10 months and 32 months, respectively (OR = 1.700, 95% CI (1.328 ~ 2.174), p < 0.001). According to the previous study's cut-off values of LDH 14, the median time to endpoint was 23 months and 16 months, respectively (OR = 2.245, 95% CI (1.021 ~ 5.990), p = 0.026). Based on the cut-off values of PLR in previous study 15, the median time to endpoint was 33 months and 29 months, respectively (OR = 2.279, 95% CI (1.180-6.606), p = 0.034). Specific grouping conditions and the median time to endpoint are shown in Table 8. The survival curve of TTN, LDH, PLR and CRPC-free survival is shown in Figure 2.

### Predictive value of TTN combined with PLR and LDH as early drug resistance biomarkers for prostate cancer

**Figure 3:**
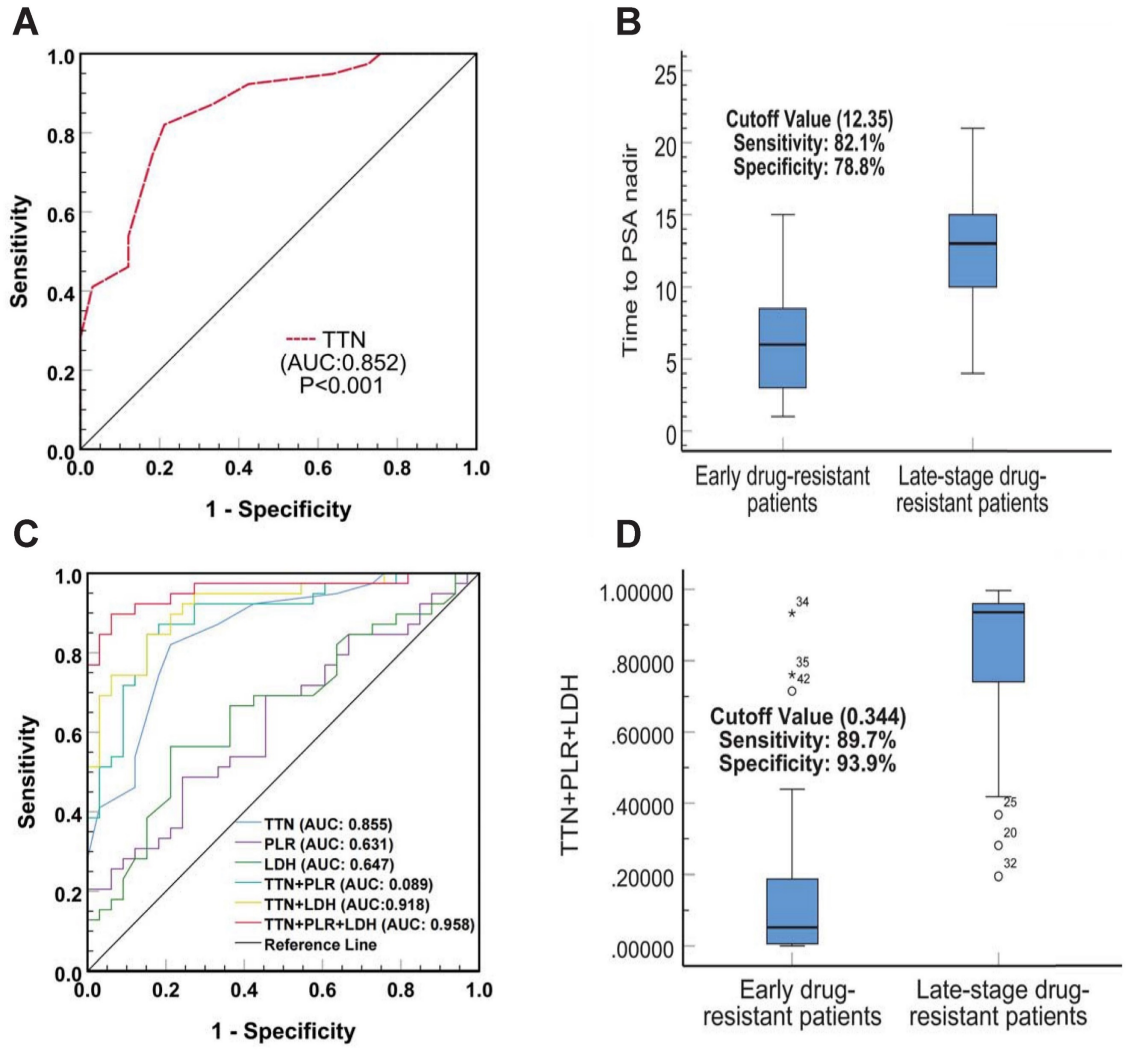
** The diagnostic value of TTN, LDH and PLR as biomarkers for prostate cancer.** The ROC curve **(A)** and cutoff value **(B)** for TTN including prostate cancer early drug-resistant patients and late-stage drug-resistant patients. The ROC curves**(C)** and cutoff value **(D)** for TTN, LDH, PLR in combination including prostate cancer early drug-resistant patients and late-stage drug-resistant patients.

To verify whether TTN has predictive value for early resistance in prostate cancer patients, we plotted Receiver Operator Characteristic (ROC) Curves to determine its threshold value. As shown in Figure 3A, TTN can easily distinguish between patients with early and late resistant prostate cancer with an area under the curve (AUC) of 0.852 (95% CI (0.768-0.942), p < 0.001). The best critical value for TTN was 12.35 months (sensitivity 82.1%, specificity 78.8%) (Figure 3B). The results showed that the predictive value of TTN was much higher than that of PLR (AUC 0.631) or LDH (0.647). In addition, the combined ROC curve for TTN and PLR, LDH showed an increased AUC value of 0.958 (95% CI (0.911-0.997), p < 0.001) (Figure 3C). The combined best critical value was 0.344 (sensitivity 89.7%, specificity 93.9%) (Figure 3D), which suggested that TTN combined with PLR and LDH can provide better predictive power for early drug resistance in prostate cancer patients.

### Correlation between clinicopathological features and TTN, LDH, and PLR

**Figure 4:**
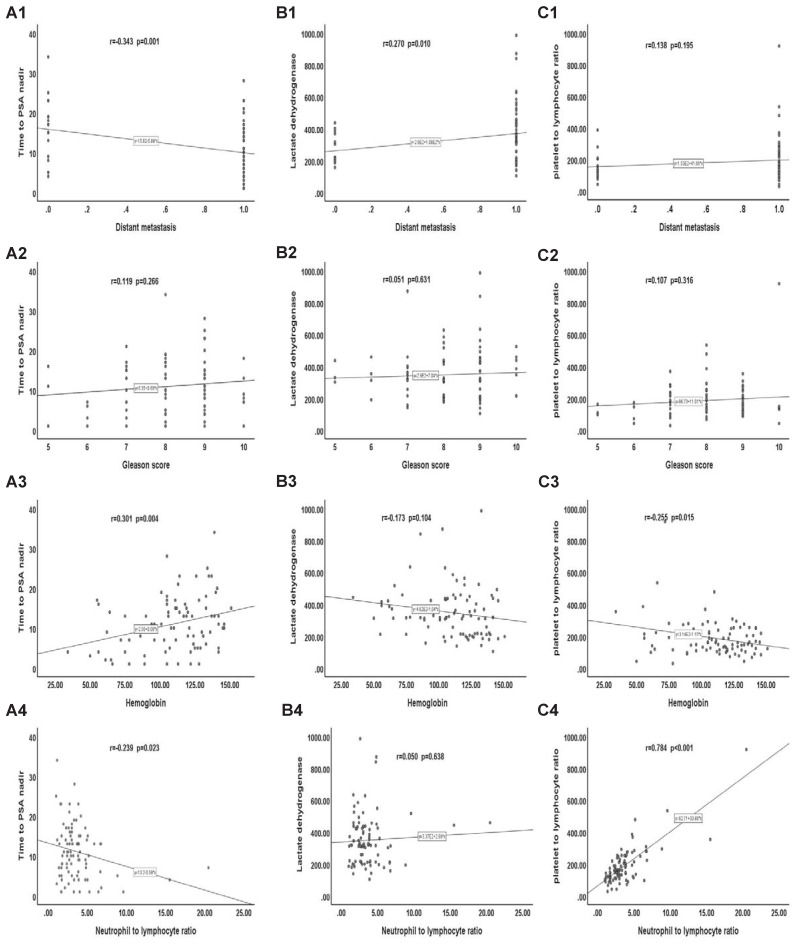
** Correlation between clinicopathological features and TTN、LDH and PLR. (A)** Correlation of clinicopathological features with TTN. **(B)** Correlation of clinicopathological features with LDH. **(C)** Correlation of clinicopathological features with PLR.

We analyzed the correlation between clinicopathological features and TTN, LDH, and PLR. Our results showed that distant transfer was inversely correlated with the TTN (r = -0.343, p = 0.001). Haemoglobin was positively correlated with TTN (r = 0.301, p = 0.004). There was a positive correlation between NLR and PLR (r = 0.784, p < 0.001). However, no significant correlation was found between distant transfer and LDH, PLR, or between GS score and TTN, LDH, and PLR, or between haemoglobin and LDH, PLR, or between NLR and TTN, LDH (Figure 4).

## Discussion

In fact, many prostate cancer patients have already been in the middle or advanced stage of the disease at the time of treatment and thus miss the opportunity for radical surgery. At this time, endocrine therapy can be regarded as one of the best treatment options. Clinical observation has found that, although endocrine treatment can improve a patient's overall survival rate (OS), its long-term effects vary greatly 16. Because the occurrence mechanism of CRPC will be extraordinarily complex and has no effective treatment 17, once the patient advances to the CRPC stage, treatment is challenging, and the prognosis is inferior. Therefore, we judged the forecast by analyzing the risk factors that may affect early progression to CRPC (within 18 months after endocrine treatment) and using this to guide treatment.

In comparing the sensitive and drug-resistant groups, we found that the shorter the TTN, the more likely prostate cancer was to become CRPC (p < 0.031). TTN was defined as the time from initiation of endocrine to the nadir of PSA level during the treatment. As one of the originators of the concept, Cooper EH argued that TTN is closely related to prostate cancer progression 18. Sasaki's study showed that the longer TTN and lower PSA nadir means, the lower risk of prostate cancer progression 19. Unlike Sasaki's study, the PSA nadir value showed significant differences in our univariate test (p = 0.003), but there was no statistically significant difference in our multivariate analysis (p = 0.999). This may be due to spurious associations between numerous exposures and outcomes (development of CRPC). Our results suggest that TTN is more associated with CRPC progression than changes in PSA values, but we cannot exclude the possibility of a single center or small sample size.

We found that LDH levels are significantly increased in various malignant tumours, and the relevant literature shows that LDH plays a crucial role in tumour metabolism, proliferation, invasion and metastasis 20. Some studies have shown that LDH levels have significant prognostic value for many solid tumours, such as liver cancer and renal clear cell carcinoma 21, 22. The results of our study confirmed that the higher the serum LDH before endocrine treatment, the greater the possibility of the progression of CRPC. Indeed, From the 2004 year when Halabi included LDH as a prognostic model for predicting survival in metastatic CRPC patients 23, to the recent continuously updated and adjusted of this model by Armstrong et al. 14, many studies have provided sufficient evidence for the prognostic value of LDH in patients with CRPC. Furthermore, recent studies by Heck have also demonstrated that in treating prostate cancer patients with organ metastasis, elevated LDH is related to a worse prognosis for prostate cancer patients with organ metastases 24.

In the comparison between the early and late drug-resistant group, we also found that the correlation between TTN and the onset of CRPC was statistically significant. That is the shorter the TTN, the faster the time for the progression of CRPC. Research has shown that the dynamic change index of PSA is a standard indicator used to predict the time of CRPC progression, and the widely accepted view is that the faster the rate of PSA decline, the better the prognosis 25. In recent years, more evidence has suggested that longer TTN was closely associated with more extended periods of progression-free survival (PFS)^26^, ^27^. Morote et al. found that the faster the PSA decreased to the lowest value, the shorter the PFS and OS 28. The value of TTN > 12 months after ADT is reported to be the most important early predictor of prolonged survival in prostate cancer patients with bone metastasis 29. Although most clinicians generally believe that a sharp decline in PSA is always associated with a better response, our study suggests that a more extended time taken to achieve a PSA nadir may represent a state of sustained androgen sensitivity following ADT treatment. However, the decline in PSA in a short period may be due to endocrine therapy inhibiting androgen receptor-mediated PSA expression levels in HSPC cells. A shorter time to reach the nadir PSA level and faster death of HSPC cells may induce the growth and proliferation of hormone-resistant prostate cancer cells, leading to the progression of CRPC 17.

Meanwhile, we also found that higher serum LDH levels before endocrine therapy suggested a shorter progression to CRPC. The study showed that higher LDH level before ADT was closely related to poor OS and PFS, and subgroup analysis confirmed that increased LDH baseline levels accelerated disease progression in CRPC and HSPC patients 30. The previous study also confirmed this, in which among the 442 CRPC patients included, the median OS was 40.7 months (95% CI; 36.8-44.0), and the median OS in patients with elevated LDH was only 30.6 months (95% CI 27.6-36.5) 31. Heller et al. also found that the combination of LDH with other biomarkers (e. g., PSA or CTC counts) also provides evidence for improving the risk stratification of disease and extending the period of disease progression 32. The specific mechanism of the role of LDH in tumour progression is unclear. The massive production of lactic acid leads to increased local acidity in the tumour microenvironment, which is harmful to normal cells and beneficial for tumour cells. Therefore, at the early hormone-sensitive stage, the mechanism may be associated with a reversible reaction of lactic acid dehydrogenase catalyzing the conversion of pyruvate to lactate, which plays a crucial role in the glycolysis of tumour cells 33.

Furthermore, our study found that a higher PLR before endocrine therapy suggested a shorter time for progression to CRPC. PLR, a biomarker of the systemic inflammatory response, has been shown to have a predictive value in assessing the presence and progression of cancer, such as ovarian cancer and colorectal cancer, and in response to drug therapy 34, 35. Recently, there has also been more evidence that inflammation may play an essential role in the development and progression of prostate cancer 36, 37. Wang et al. found that high PLR is an independent prognostic factor for PFS and OS in prostate cancer ADT treatment and could predict a poor prognosis in patients with a GS > 7 or bone metastases 37. The difference between our study and Wang et al. is that there is no difference in PLR between the drug-sensitive group and the drug-resistant group, but there is a significant difference in PLR between the early-stage and late-stage drug-resistant groups, which may be related to the insensitive response of PLR to low tumour load. The mechanisms underlying our observations were unclear. The elevated PLR reflects an elevated platelet-dependent pro-tumour response 38 and a reduced lymphocyte-mediated antitumor immune response 39, which may contribute to cancer progression and adverse outcomes. Currently, there are few studies on the prognosis of PLR in the ADT treatment of prostate cancer. To our knowledge, this is the first time PLR has been shown to be associated with early progression in HSPC patients.

In our study, p63 expression was higher in HSPC than in CRPC, and this expression was statistically different. P63 (encoded by TP63) is a member of the p53 family of transcription factors and is a prostate basal cell marker 40. It is precise because of the lack of basal cells in prostate adenocarcinoma and the low expression of p63 that immunostaining for p63 is often used to distinguish prostate cancer from benign masses 41. Although p63 is usually lowly expressed in the cytoplasmic staining of prostate cancer cells, it has been found that p63 is also aberrantly and diffusely expressed 42. Further exploration of P63 in prostate cancer revealed a negative correlation between abnormal p63 expression in prostate tumour tissue at diagnosis and prostate cancer progression 43,44, which is consistent with our findings. However, controversy remains regarding the relationship between the differential expression of p63 and prostate cancer-specific mortality. The opposite view to ours was reported by Dhillon et al. 45. Dhillon explained this discrepancy by the possibility that they had discovered a rare phenomenon 42 or that the misalignment and imbalance of the p63 heterodimer may have altered the stability and function of p63, thereby disrupting cell cycle arrest and apoptosis and ultimately promoting prostate cancer progression. In conclusion, we found that the high expression of p63 before ADT, although not associated with the early or late progression of CRPC, clearly suggested that the prognosis of prostate cancer patients would be relatively better.

Finally, by comparing the survival curves of a different group of CRPC patients, we found that TTN, LDH, and PLR not only have value for the prognostic of CRPC at both time intervals of 24 and 18 months but also play a role in CRPC PFS. The ROC curve showed that the predictive value of TTN (AUC 0.852) (95% CI 0.768-0.942, p < 0.001) was much higher than that of PLR (AUC 0.631) or LDH (AUC 0.647) and that the AUC value of TTN combined with PLR and LDH increased to 0.958 (95% CI 0.911-0.997, p < 0.001). Moreover, our correlation analysis also found that TTN was inversely correlated with the tumour M stage (r = -0.343; p = 0.001) and positively associated with haemoglobin (r = 0.301; p = 0.004). There was a positive correlation between PLR and NLR (r = 0.784; p < 0.001). This study has certain limitations. First, our study was conducted at a single institution with a small sample size of 90 patients with a short-term follow-up time of 3 to 60 months and a mean follow-up time of 24.75 months. It has been shown that NLR 23, a biomarker of the systemic inflammatory response, and the liver function marker AST 46, are associated with a poor prognosis of CRPC. Our study included them in our analysis, but did not find statistically significant results, which may account for the relatively small number of cases. Therefore, to provide better evidence, our results require testing with a larger sample, preferably a multicenter cohort. Second, the data collected from HIS system only covered part of the available clinical and pathological information, and relevant immunohistochemical data and other biomarker information (such as circulating tumour cells, etc.) were not included in the analysis. Finally, a longitudinal analysis of baseline data is lacking because of incomplete follow-up data for some patients.

In conclusion, our study shows that TTN combined with LDH and PLR can better predict the early (18 months) progression of HSPC to the CRPC stage. p63 expression is associated with favorable prognosis in prostate cancer patients.

## Figures and Tables

**Table 1:** Baseline clinical characteristics of patients treated with androgen deprivation therapy.

Variables	Group A (n=18)	Group B (n=72)	P value
**Age (y)**	70.72±8.94	70.67±9.64	0.888
**PSA on initiation of ADT (ng/ml)**	15.28±6.80	478.17±796.73	<0.001*
**PSA nadir (ng/ml)**	0.05±0.10	18.02±36.40	<0.001*
**TTN (m)**	19.50±5.79	8.93±5.15	<0.001*
**PSA decline velocity (ng/ml·month)**	0.85±0.48	157.61±422.29	<0.001*
**Tumor Stage**			0.063
**1-2**	6	3	
**3**	9	40	
**4**	3	29	
**Lymphatic metastasis**			0.002*
**0**	11	31	
**1**	7	41	
**Distant metastasis**			<0.001*
**0**	10	7	
**1**	8	65	
**Osseous metastasis**			0.008*
**0**	13	27	
**1**	5	45	
**Gleason score (n)**			0.028*
**≦7**	4	14	
**>7**	8	58	
**WBC (10^9/L)**	6.09±2.02	6.67±3.04	0.425
**RBC (10^12/L)**	4.17±1.00	3.75±0.96	0.066
**Hb (g/L)**	122.28±22.96	106.64±26.32	0.013*
**PLT (10^9/L)**	217.64±52.51	235.42±100.39	0.443
**HCT (%)**	37.91±6.49	33.05±11.18	0.003*
**MCV (fL)**	94.37±6.15	91.84±17.91	0.029*
**MCH (pg)**	30.71±2.84	29.02±3.83	0.043*
**MCHC (g/L)**	323.18±14.80	323.25±12.81	0.940
**ANC (10^9/L)**	3.98±1.29	4.71±2.84	0.480
**LY (10^9/L)**	1.46±0.81	1.41±0.48	0.747
**PLR**	185.71±87.72	187.31±125.97	0.628
**NLR**	3.32±1.60	3.76±2.97	0.956
**MONO (10^9/L)**	0.47±0.22	0.51±0.20	0.220
**PCT (ug/L)**	0.21±0.05	0.23±0.09	0.582
**MPV (fL)**	10.20±0.99	9.74±0.94	0.141
**ALT (U/L)**	16.37±7.89	16.91±12.80	0.956
**AST (U/L)**	22.21±5.84	26.35±13.17	0.191
**AST/ALT**	1.65±1.00	2.28±1.52	0.023*
**LDH (U/L)**	203.78±61.48	384.03±154.43	<0.001*

*Statistically significant: P value<0.05.

**Table 2:** Univariate and multivariable analyses of risk factors of progression to CRPC after androgen deprivation therapy.

Variables	Univariate analysis		Multivariate analysis	
	OR (95% CI)	P value	OR (95% CI)	P value
**Age (y)**	0.999(0.946-1.056)	0.982		
**PSA on initiation of ADT (ng/ml)**	1.122(1.041-1.21)	0.003*	1.016(0-110518.017)	0.998
**PSA nadir (ng/ml)**	159.858(1.989-12847.619)	0.023*	2.3	0.999
**TTN (m)**	0.675(0.557-0.817)	<0.001*	0.339(0.127-0.905)	0.031*
**PSA decline velocity (ng/ml·month)**	4.749(1.314-17.166)	0.017*	0.937	0.999
**Tumor Stage**	3.372(0.896-12.698)	0.072		
**1-2**				
**3**				
**4**				
**Lymphatic metastasis**	5.179(1.728-15.522)	0.003*	722077	0.995
**0**				
**1**				
**Distant metastasis**	11.607(3.45-39.054)	<0.001*	8752730.326	0.999
**0**				
**1**				
**Osseous metastasis**	4.333(1.391-13.5)	0.011*	<0.001	0.997
**0**				
**1**				
**Gleason score (n)**	3.314(1.106-9.934)	0.032*	12270.581	0.998
**≦7**				
**>7**				
**WBC (10^9/L)**	1.089(0.876-1.353)	0.444		
**RBC (10^12/L)**	0.653(0.387-1.1)	0.109		
**Hb (g/L)**	0.197(0.066-0.587)	0.030*	0.621	0.998
**PLT (10^9/L)**	1.002(0.996-1.008)	0.466		
**HCT (%)**	0.961(0.913-1.011)	0.128		
**MCV (fL)**	0.992(0.965-1.019)	0.566		
**MCH (pg)**	0.827(0.671-1.02)	0.075		
**MCHC (g/L)**	1(0.962-1.041)	0.986		
**ANC (10^9/L)**	1.176(0.869-1.591)	0.293		
**LY (10^9/L)**	0.823(0.333-2.034)	0.673		
**PLR**	1(0.996-1.005)	0.959	0.818	0.997
**NLR**	1.076(0.846-1.37)	0.549	0.046	0.999
**MONO (10^9/L)**	2.184(0.153-31.227)	0.565		
**PCT (ug/L)**	6.597(0.013-3351.327)	0.553		
**MPV (fL)**	0.621(0.367-1.051)	0.076		
**ALT (U/L)**	1.004(0.959-1.051)	0.866	0.305	0.997
**AST (U/L)**	1.043(0.978-1.112)	0.201		
**AST/ALT**	1.715(0.896-3.282)	0.104		
**LDH (U/L)**	1.022(1.011-1.033)	<0.001*	1.065(1.014-1.119)	0.013*

*Statistically significant: P value<0.05

**Table 3:** Baseline clinical characteristics of patients in Group B.

Variables	Group B1 (n=39)	Group B2 (n=33)	P value
**Age (y)**	69.92±10.06	71.55±9.18	1.000
**PSA on initiation of ADT (ng/ml)**	553.18±905.80	389.52±647.41	0.849
**PSA nadir (ng/ml)**	27.35±46.60	6.99±11.39	0.003*
**TTN (m)**	6.05±3.87	12.33±4.36	<0.001*
**PSA decline velocity (ng/ml·month)**	270.00±553.51	34.24±53.55	0.072
**Tumor Stage**			1.000
**1-2**	2	1	
**3**	15	16	
**4**	22	16	
**Lymphatic metastasis**			1.000
**0**	18	13	
**1**	21	20	
**Distant metastasis**			0.465
**0**	2	5	
**1**	37	28	
**Osseous metastasis**			1.000
**0**	13	14	
**1**	26	19	
**Gleason score (n)**			1.000
**≦7**	8	6	
**>7**	31	27	
**WBC (10^9/L)**	7.18±3.49	6.06±2.31	0.453
**RBC (10^12/L)**	3.68±0.78	3.84±1.15	1.000
**Hb (g/L)**	103.52±28.36	110.33±23.57	1.000
**PLT (10^9/L)**	260.87±119.08	205.33±61.61	0.075
**HCT (%)**	30.85±8.45	35.65±13.41	0.402
**MCV (fL)**	87.73±8.63	96.70±24.05	0.045*
**MCH (pg)**	28.05±4.46	30.16±2.53	0.222
**MCHC (g/L)**	322.84±12.35	323.74±13.51	1.000
**ANC (10^9/L)**	5.20±3.45	4.12±1.77	0.417
**LY (10^9/L)**	1.37±0.49	1.45±0.48	1.000
**PLR**	216.15±156.25	153.22±63.26	0.171
**NLR**	4.42±3.81	2.98±1.11	0.570
**MONO (10^9/L)**	0.52±0.24	0.48±0.13	0.993
**PCT (ug/L)**	0.25±0.11	0.20±0.06	0.045*
**MPV (fL)**	9.83±1.00	9.64±0.88	1.000
**ALT (U/L)**	17.39±14.52	16.34±10.60	1.000
**AST (U/L)**	26.55±14.49	26.11±11.63	1.000
**AST/ALT**	2.55±1.84	1.97±0.96	0.588
**LDH (U/L)**	425.77±177.22	334.70±104.83	0.066

*Statistically significant: P value<0.05; P value was adjusted.

**Table 4:** Univariate and multivariable analyses of risk factors of progression to CRPC in Group B.

Variables	Univariate analysis		Multivariate analysis	
	OR (95% CI)	P value	OR (95% CI)	P value
**Age (y)**	0.982(0.935-1.032)	0.475		
**PSA on initiation of ADT (ng/ml)**	1(1-1.001)	0.388		
**PSA nadir (ng/ml)**	1.042(1.006-1.079)	0.023*	1.027(0.979-1.078)	0.269
**TTN (m)**	0.703(0.599-0.825)	<0.001*	0.548(0.441-0.732)	<0.001*
**PSA decline velocity (ng/ml·month)**	1.006(0.999-1.012)	0.084		
**Tumor Stage**	1.352(0.523-3.499)	0.739		
**1-2**				
**3**				
**4**				
**Lymphatic metastasis**	1.292(0.399-4.184)	0.670		
**0**				
**1**				
**Distant metastasis**	3.304(0.596-18.297)	0.303		
**0**				
**1**				
**Osseous metastasis**	1.474(0.565-3.845)	0.428		
**0**				
**1**				
**Gleason score (n)**	0.861(0.265-2.795)	0.803		
**≦7**				
**>7**				
**WBC (10^9/L)**	1.158(0.957-1.401)	0.007*	1.062(0.432-2.611)	0.896
**RBC (10^12/L)**	0.84(0.514-1.373)	0.488		
**Hb (g/L)**	0.99(0.972-1.008)	0.274		
**PLT (10^9/L)**	1.007(1.001-1.012)	0.026*	0.989(0.960-1.019)	0.469
**HCT (%)**	0.95(0.895-1.009)	0.093		
**MCV (fL)**	0.891(0.81-0.979)	0.016*	0.975(0.886-1.074)	0.614
**MCH (pg)**	0.812(0.673-0.98)	0.030*	0.861(0.671-1.105)	0.241
**MCHC (g/L)**	0.994(0.959-1.031)	0.764		
**ANC (10^9/L)**	1.198(0.948-1.513)	0.131		
**LY (10^9/L)**	0.677(0.253-1.811)	0.437		
**PLR**	1.006(1-1.013)	0.044*	1.016(1.015-1.028)	0.005*
**NLR**	1.335(0.986-1.809)	0.062	0.688(0.188-2.523)	0.573
**MONO (10^9/L)**	2.887(0.255-32.708)	0.392		
**PCT (ug/L)**	1662.657(4.154-665493.481)	0.015*	1.000(0.918-1.089)	0.998
**MPV (fL)**	1.241(0.743-2.074)	0.409		
**ALT (U/L)**	1.007(0.97-1.045)	0.728	1.012(0.994-1.086)	0.737
**AST (U/L)**	1.003(0.968-1.039)	0.886		
**AST/ALT**	1.353(0.915-2)	0.130		
**LDH (U/L)**	1.005(1.001-1.009)	0.019*	1.018(1.007-1.029)	0.001*

*Statistically significant: P value<0.05.

**Table 5:** Kruskal-Wallis H test for HSPC patients compared with CRPC patients.

Variables	Group Avs B1		Group Avs B2	
	H value	P value	H value	P value
**PSA**	-43.013	<0.001*	-35.309	<0.001*
**PSA nadir**	-49.222	<0.001*	-29.619	<0.001*
**TTN**	52.035	<0.001*	22.358	<0.001*
**PSA declin velocity**	-46.260	<0.001*	-31.533	<0.001*
**MCV**	22.395	0.024*	6.735	1.000
**LDH**	-41.037	<0.001*	-28.768	<0.001*

*Statistically significant: P value<0.05. P value was corrected; Only statistically significant variables were listed here.

**Table 6:** The expression of immunohistochemical indexes within different groups.

Variables	Group A (n=18)	Group B1 (n=39)	Group B2 (n=33)
**AR**	15/18=83.33%	36/39=92.30%	31/33=93.94%
**PSA**	16/18=88.89%	35/39=89.74%	26/33=78.79%
**PSAP**	17/18=94.44%	37/39=94.87%	30/33=90.91%
**CK**	15/18=83.33%	29/39=74.36%	25/33=75.76%
**P504s**	15/18=83.33%	31/39=79.49%	29/33=87.89%
**p63**	9/18=50%	5/39=12.82%	3/33=9.09%

**Table 7:** Chi-square test for immunohistochemical indexes between different groups.

Variables	GroupAvs B		GroupAvs B1		GroupAvs B2		GroupB1vs B2	
	χ^2^ value	P value	χ^2^ value	P value	χ^2^ value	P value	χ^2^ value	P value
**AR**	1.681	0.195	1.053	0.305	1.482	0.224	0.074	0.786
**PSA**	0,202	0.653	0.010	0.992	0.818	0.366	1.658	0.198
**PSAP**	0.045	0.833	0.005	0.947	0.201	0.654	0.434	0.510
**CK**	0.447	0.504	0.563	0.453	0.395	0.530	0.019	0.891
**P504s**	0.001	1.000	0.117	0.732	0.203	0.652	0.906	0.341
**p63**	14.214	0.001*	9.188	0.002*	10.833	0.001*	0.252	0.616

*Statistically significant: P value<0.05.

**Table 8:** Log-rank test for final independent predictors.

Variables	Group A (n=18)	Group B (n=72)	Median time to endpoint / months	P value	Group B1 (n=39)	Group B2 (n=33)	Median time to endpoint / months	P value
**TTN (months)**				<0.001*				<0.001*
**≤ 12**	0	39	16		32	7	10	
**> 12**	18	33	45		7	26	32	
**LDH (U/L)**				<0.001*				0.026*
**≤ 300**	16	14	43		5	9	23	
**> 300**	2	58	16		34	24	16	
**PLR**				0.191				0.034*
**≤ 132**	5	26	45		11	15	33	
**> 132**	13	46	43		28	18	29	

*Statistically significant: P value<0.05.

## Abbreviations

CRPC: castration-resistant prostate cancer
ADT: androgen deprivation therapy
HIS: health information system
HSPC: hormone-sensitive prostate cancer
PSA: prostate-specific antigen
TTN: time to PSA nadir
GS: gleason score
WBC: white blood cell
RBC: red blood cell
Hb: haemoglobin
PLT: platelets
HCT: hematocrit
MCV: mean corpuscular volume
MCH: mean erythrocyte haemoglobin content
MCHC: mean corpuscular haemoglobin concentration
ANC: absolute neutrophil count
LY: lymphocyte count
PLR: platelet to lymphocyte ratio
NLR: neutrophil to lymphocyte ratio
MONO: monocyte count
PCT: procalcitonin
MPV: mean platelet volume
ALT: alanine transaminase
AST: aspartate amino transferase
LDH: lactate dehydrogenase
OS: overall survival
PFS: progression-free survival
AUC: area under the curve
ROC: receiver operator characteristic
